# Cytochrome P450 CYP71BE5 in grapevine (*Vitis vinifera*) catalyzes the formation of the spicy aroma compound (−)-rotundone

**DOI:** 10.1093/jxb/erv496

**Published:** 2015-11-20

**Authors:** Hideki Takase, Kanako Sasaki, Hideyuki Shinmori, Akira Shinohara, Chihiro Mochizuki, Hironori Kobayashi, Gen Ikoma, Hiroshi Saito, Hironori Matsuo, Shunji Suzuki, Ryoji Takata

**Affiliations:** ^1^Laboratory, New Product & Process Developments, Mercian Corporation, 4-9-1 Johnan, Fujisawa, Kanagawa 251-0057, Japan; ^2^The Institute of Enology and Viticulture, University of Yamanashi, 1-13-1 Kitashin, Kofu, Yamanashi 400-0005, Japan; ^3^Interdisciplinary Graduate School of Medical and Engineering, University of Yamanashi, 4-4-37 Takeda, Kofu, Yamanashi 400–8510, Japan; ^4^Château Mercian,1425-1 Shimoiwasaki, Katsunuma, Koshu, Yamanashi 409–1313,Japan

**Keywords:** Cytochrome P450, grapevine, guaiene, rotundone, sesquiterpene oxidase, *Vitis vinifera*, wine.

## Abstract

(−)-Rotundone is a potent odorant molecule with a characteristic spicy aroma existing in various plants including grapevines (*Vitis vinifera*). It is considered to be a significant compound in wines and grapes because of its low sensory threshold and aroma properties. (−)-Rotundone was first identified in red wine made from the grape cultivar Syrah and here we report the identification of VvSTO2 as a α-guaiene 2-oxidase which can transform α-guaiene to (−)-rotundone in the grape cultivar Syrah. It is a cytochrome P450 (CYP) enzyme belonging to the CYP 71BE subfamily, which overlaps with the very large CYP71D family and, to the best of our knowledge, this is the first functional characterization of an enzyme from this family. *VvSTO2* was expressed at a higher level in the Syrah grape exocarp (skin) in accord with the localization of (−)-rotundone accumulation in grape berries. α-Guaiene was also detected in the Syrah grape exocarp at an extremely high concentration. These findings suggest that (−)-rotundone accumulation is regulated by the *VvSTO2* expression along with the availability of α-guaiene as a precursor. *VvSTO2* expression during grape maturation was considerably higher in Syrah grape exocarp compared to Merlot grape exocarp, consistent with the patterns of α-guaiene and (−)-rotundone accumulation. On the basis of these findings, we propose that VvSTO2 may be a key enzyme in the biosynthesis of (−)-rotundone in grapevines by acting as a α-guaiene 2-oxidase.

## Highlight

CYP71BE5 from grapevine was identified as a sesquiterpene oxidase capable of transforming α-guaiene to (−)-rotundone, responsible for the characteristic spicy aroma in wines.

## Introduction

Wine flavor is complex, involving hundreds of volatile molecules. Among them, only a few molecules have been identified as valuable contributors to wine aroma, such as monoterpenoids (Rapp and Mandery, 1986), C13 norisoprenoids (Winterhalter and Rouseff, 2002), volatile sulfur compounds (Darriet *et al.*, 1995; Tominaga *et al.*, 1996, 1998a) and methoxypyrazines (Allen *et al.*, 1991). These compounds mainly originate from the secondary metabolites produced in grapevines (*Vitis vinifera*). Some of them exist as odorless forms, such as cysteine (Tominaga *et al.*, 1998b; Thibon *et al.*, 2010), glutathione (Peyrot Des Gachons *et al.*, 2002) and glycoside conjugates (Park *et al.*, 1991) and can be released by chemical and enzymatic reactions during wine making and aging. In addition, the relative concentrations of these compounds in grape berries depend on the grape variety and are further influenced by diverse factors such as environmental conditions and the timing of harvest (Styger *et al.*, 2011). Finally, the combinations of these compounds can be expressed by wine makers as the attractive characteristics of each wine, reflecting the grape variety and *terroir*.

(−)-Rotundone, an oxygenated sesquiterpene, has been identified as a molecule responsible for the spicy aroma in various plants including grapes and a large number of important herbs and spices such as black and white pepper, oregano, basil, thyme, marjoram and rosemary (Wood *et al.*, 2008). Since this molecule is an extremely potent aroma compound with a low sensory threshold (16ng l^-1^ in red wine, 8ng l^-1^ in water), even trace levels of (−)-rotundone can give a pleasant peppery aroma to various foods and beverages, including wine. (−)-Rotundone has been found in several grape varieties, particularly Syrah (regionally called Shiraz), Mourvèdre, and Durif from Australia (Wood *et al.*, 2008; Herderich *et al.*, 2012), and Schioppettino, Vespolina, and Grüner Veltliner from Europe (Caputi *et al.*, 2011; Mattivi *et al.*, 2011). It has been also detected in several tissues, especially the exocarp, stem and leaf tissues (Capone *et al.*, 2012). Furthermore, the accumulation of (−)-rotundone during grape maturation is influenced by diverse environmental factors, such as low atmospheric temperatures (Caputi *et al.*, 2011; Herderich *et al.*, 2012), soil properties and topography (Scarlett *et al.*, 2014), and soil moisture from irrigation (Geffroy *et al.*, 2014). A recent study showed that a grape surface temperature exceeding 25°C negatively affects the concentration of (−)-rotundone in Syrah grapes (Zhang *et al.*, 2015). Thus, (−)-rotundone is increasingly the focus of much interest as an aroma. However, the mechanism of its biosynthesis in plants is still unclear.

α-Guaiene is a sesquiterpene hydrocarbon found in oil extracts from various plants (Kapadia *et al.*, 1967; Rakotonirainy *et al.*, 1997; Pino *et al.*, 2001) including grapevines (Schreier *et al.*, 1976; Coelho *et al.*, 2006). This compound is a potential precursor of (−)-rotundone, as they have common structures, namely, the unique five- and seven-membered rings. Therefore, (−)-rotundone may be synthesized by the oxidation of α-guaiene at position carbon-2(C-2). The chemical formation of (−)-rotundone by the aerial oxidation of α-guaiene via 2-hydroperoxyguaiene has been proposed (Huang *et al.*, 2014) and (2R)-rotundol and (2S)-rotundol are reported intermediates. (Huang *et al.*, 2015a, b). This type of chemical formation may be one of the pathways to synthesize (−)-rotundone. However, further investigation is required to explain the mechanisms of the formation of rotundone in grapevines in terms of oxidation by the enzymes.

Cytochrome P450 (CYP) enzymes play critical roles in oxidative reactions during the biosynthesis of various natural compounds in plants including terpenoids. Several CYPs, for example the (+)-δ-cadinene-8-hydroxylase CYP706B1 (Luo *et al.*, 2001), the premnaspirodiene oxygenase CYP71D55 (Takahashi *et al.*, 2007), the α-humulene 10-hydroxylase CYP71BA1 (Yu *et al.*, 2011), the santalene/bergamotene oxidase CYP76F39v1, the bergamotene oxidase CYP76F37v1 (Diaz-Chavez *et al.*, 2013) and the (+)-valencene oxidase CYP71AV8 (Cankar *et al.*, 2011), have been reported to catalyze the oxidation of sesquiterpenes in several plants. However, plant CYPs that can transform α-guaiene to (−)-rotundone have never been reported.

Here, we identify the α-guaiene 2-oxidase VvSTO2, which is capable of transforming α-guaiene to (−)-rotundone, from the grapevine cultivar Syrah. It is a cytochrome P450 belonging to the CYP71BE subfamily. Finally, we propose that VvSTO2 is one of the key enzymes involved in the pathway of (−)-rotundone biosynthesis in grapevines.

## Materials and methods

### Plant materials and chemicals

Grapevines, *Vitis vinifera*, were grown in Nagano Prefecture (lat. 36º20′35′′N; long. 138º18′5′′E; 640 m above sea level) in Japan. The cultivars Syrah and Merlot were harvested in the growing season of 2012/13.

(−)-Rotundone and deuterated ^2^H_5_-rotundone were prepared as previously described (Takase *et al.*, 2015). α-Guaiene was synthesized from guaiol by a dehydration procedure with thionyl chloride as the middle step of (−)-rotundone synthesis, and purified by thin layer chromatography using Merck silica gel 60F-254 plates (0.25mm, precoated). α-Guaiene [purity, 90% by gas chromatography-mass spectrometry (GC-MS) analysis] was identified by nuclear magnetic resonance (NMR) spectroscopy as described previously (Ito *et al.*, 2005). β-Nootkatol was synthesized by the reduction of nootkatone as previously reported (Takahashi *et al.*, 2007) with slight modification and identified by NMR spectroscopy. ^1^H NMR (400 MHz) and ^13^C NMR (100MHz) spectra were acquired with a Bruker AVANCE 400 spectrometer and measurements were conducted in deuterated chloroform (CDCl_3_) at ambient temperature. Chemical shifts were recorded in parts per million (ppm). All chemicals required for the synthesis, CDCl_3_ for NMR, HPLC-grade ethanol, HPLC-grade methanol, n-hexane, n-pentane, ethyl acetate, (−)-α-cedrene and (+)-valencene, were purchased from Sigma-Aldrich Japan (Osaka, Japan). Milli-Q water was obtained from a Milli-Q purification system (Merck Millipore, Tokyo, Japan).

### Isolation of CYPs from *V. vinifera* cv. Syrah

Total RNA was extracted from the Syrah grape exocarp by the cetyltrimethylammonium bromide (CTAB) method (Chang *et al.*, 1993), treated with an RNase-Free DNase set (Qiagen, Valencia, CA, USA) and purified with an RNeasy Plant Mini kit (Qiagen) in accordance with the provided protocol. cDNA was synthesized using SuperScript III reverse transcriptase (Life Technologies Inc., Rockville, MD, USA). To obtain the full-length cDNA, polymerase chain reaction (PCR) was performed using Phusion High-Fidelity DNA Polymerase (New England BioLabs Inc., Ipswich, MA, USA) with cDNA as the template. Gene-specific primers were designed with the information in the 12-fold coverage genome sequence assembly of the grapevine cultivar Pinot Noir PN40024 (NCBI BioProject, accession: PRJNA33471) for the target gene, primer 1 and 2 originating from XM_010646246 for *VvSTO2*, primer 3 and 4 originating from XM_010654905 for *VvSTO4,* and primer 5 and 6 originating from XM_010657579 and NC_012016 for *VvSTO6*, as listed in Supplementary Table S1 at *JXB* online. PCR products of ~1.5kb were subcloned into the pT7 Blue vector via TA cloning using a DNA ligation kit (Takara Bio Inc., Japan) and confirmed by sequencing.

### Heterologous expression of CYPs in yeasts

To generate C-terminal fusion proteins with the V5 epitope, cDNA fragments with each stop codon removed were amplified by PCR using primer 7 and 8 for *VvSTO2*, primer 9 and 10 for *VvSTO4,* and primer 11 and 12 for *VvSTO6* (Supplementary Table S1), and cloned into the pYES2.1/V5-His-TOPO^®^ vector via a pYES2.1 TOPO^®^ TA expression kit (Invitrogen) in accordance with the provided protocol. The resulting construct and the empty pYES2 vector (Invitrogen) as a control were transformed to the BJ2168 yeast strain (*MATa, prc1-407, prb1-1122, pep4-3, leu2, trp1, ura3-52, gal2*; Nippon Gene) as previously described (Gietz and Schiestl, 2007).

### Preparation of recombinant CYP proteins

Transformed yeast cells were grown from single colonies and incubated overnight at 30ºC with shaking in 50ml of SD medium without uracil containing 2% glucose. Yeast cells were collected by centrifugation and transferred in 250ml of SD medium without uracil containing 2% galactose at a starting OD_600_ of 0.4 to induce protein expression. After incubation overnight at 30ºC with shaking, the cells were pelleted by centrifugation. Recombinant proteins were extracted from yeast cells by the enzymatic breaking method (Pompon *et al.*, 1996) with slight modifications. Microsomes were isolated by ultracentrifugation at 100000×*g* for 60min and protein concentration was determined by the Bradford method (Coomassie Plus Protein Assay, Thermo Scientific, Rockford, IL, USA). The prepared proteins (10 μg per lane) were separated by SDS-PAGE on 10% (w/v) slab gels (e-PAGEL, ATTO Co., Tokyo, Japan) and transferred to a PVDF membrane (Amersham). Recombinant proteins were detected by western blotting with a mouse monoclonal anti-V5-HRP antibody (Invitrogen) and an ECL Prime Western Blotting Detection Reagent (Amersham).

### *In vitro* enzyme assay

Standard enzyme assays were performed in a total volume of 200 μl containing 50mM Tris-HCl, pH 7.5, 1mM NADPH, 200 μg of microsomal proteins and 100 μM substrates. The mixture was then incubated at 30ºC for 2h. The reaction products were extracted with the same volume (200 μl) of ethyl acetate. The organic phase was evaporated by N_2_ purging and directly subjected to GC-MS analysis. For determination of kinetic properties, an enzyme reaction was performed in triplicate at each substrate concentration from 2.6–130 μM of α-guaiene or from 2–100 μM of (+)-valencene with 200 μg of microsomal protein at 30ºC for 30min. Enzyme assays adding α-guaiene and (+)-valencene simultaneously at the same concentrations of 80 μM were performed in triplicate with 200 μg of microsomal protein at 30ºC for 30min, to detect whether these substrates interfere with each enzyme reaction of VvSTO2. The reaction was terminated by adding 200 μl of methanol and 10ng μl^-1^ (−)-α-cedrene as an internal standard. The resulting reaction products were diluted up to 2ml with Milli-Q and subjected to stir bar sorptive extraction (SBSE) and GC-MS analysis. Other potential substrates were purchased from Sigma-Aldrich Japan (Osaka, Japan) and tested. The kinetic properties were calculated from the Hanes-Woolf plot.

### GC-MS analysis

The *in vitro* reaction products extracted directly using ethyl acetate were analyzed on an Agilent 6890 Series GC system coupled to an Agilent 5973 insert mass selective detector (MSD). The reaction products were separated on a BP20 column (50 m × 0.22mm × 0.25 μm; Trajan Scientific and Medical, Melbourne, Australia) with helium carrier gas at a constant flow rate of 1.1ml min^-1^. The GC oven temperature was programmed from 40ºC (held for 2min) to 130ºC at 15ºC min^-1^ and then increased to 260ºC (held for 5min) at 10ºC min^-1^. MS measurements were performed in the scan mode using electron ionization at 70eV. The MS transfer line was held at 260ºC and the scan range was set from m/z 40 to 300.

### Stir bar sorptive extraction (SBSE)

SBSE was performed as previously described (Takase *et al.*, 2015). A stir bar coated with 24 μl of polydimethylsiloxane (PDMS, Twister, GERSTEL GmbH, Mülheim an der Ruhr, Germany) was conditioned for 60min at 300ºC in a flow of nitrogen. The collected grape samples were transferred to 10ml headspace vials, and the vials were sealed with a screw cap after the addition of a stir bar. SBSE was performed at room temperature for 60min by stirring at 1500rpm. The stir bar was washed with Milli-Q water, dried with lint-free paper and introduced to a glass thermal desorption liner. The glass liner was placed in a thermal desorption unit. The stir bar was reconditioned by soaking in methanol:dichloromethane (1:1, v/v) for 24h, dried at room temperature and stored carefully until the next use.

### GC-MS analysis using SBSE method

The *in vitro* reaction products obtained by SBSE were analyzed on an Agilent 7890A Series GC system equipped with an Agilent 5975C inert XL MSD with a Triple-Axis Detector, a thermal desorption unit (TDU, GERSTEL GmbH) and a programmed temperature vaporizing injector (CIS4, GESTER GmbH). For thermal desorption, the TDU was programmed from 40ºC (held for 0.5min) to 300ºC (held for 3min) at 200ºC min^-1^ with 50ml min^-1^ desorption flow. The transfer capillary temperature was fixed at 300ºC. Desorbed compounds were trapped at 10ºC on a Tenax TA packed liner in the CIS4. After desorption, the CIS4 was programmed from 10ºC to 250ºC (held for 1min) at 720ºC min^-1^ to inject trapped compounds onto the column in the splitless mode for a splitless time of 2min. The reaction products were separated on a BP20 column (50 m × 0.22mm × 0.25 μm; Trajan Scientific and Medical, Melbourne, Australia) and measured using the same conditions as described for ‘GC-MS analysis’ above.

### GC-MS/MS analysis using SBSE method

The samples collected from grape tissues by SBSE were analyzed on an Agilent 7890A Series GC system coupled to an Agilent 7000 GC/MS Triple Quad system and equipped with a TDU and a CIS4 system. For thermal desorption, the TDU and CIS4 were programed under the same conditions as for GC-MS analysis, as described above. Compounds were separated on a BP20 column (50 m × 0.22mm × 0.25 μm; Trajan Scientific and Medical) with helium carrier gas at a constant flow rate of 1.1, and the GC oven temperature was programmed from 40ºC (held for 2min) to 130ºC at 15ºC min^-1^, then increased to 160ºC at 2ºC min^-1^, and further increased to 260ºC (held for 5min) at 5ºC min^-1^. The GC/MS Triple Quad system was operated in the electron impact (EI) ionization mode at 70eV, the nitrogen flow in the collision cell was 1.5ml min^-1^ and the helium quench flow was 2.25ml min^-1^. MS measurements were performed in the multiple reaction monitoring (MRM) mode. The transitions m/z 204 to 147 for α-guaiene, m/z 218 to 163 for (−)-rotundone and m/z 223 to 166 for ^2^H_5_-rotundone as an internal standard were selected. Collision energies for α-guaiene, (−)-rotundone, and ^2^H_5_-rotundone were 3, 8 and 7eV, respectively.

### Quantitative real-time RT-PCR analysis

Total RNA was extracted from the grape exocarp and mesocarp by the CTAB method (Chang *et al.*, 1993), treated with the RNase-Free DNase Set (Qiagen) and purified with the RNeasy Plant Mini Kit (Qiagen) in accordance with the provided protocol. Quantitative real-time RT-PCR was performed with a One-Step SYBR PrimeScript PLUS RT-PCR kit (Takara-Bio Inc.) using an ABI Prism 7300 real-time PCR system (Life Technologies Inc.) in accordance with the provided protocol. The specific primers for *VvSTO2* (primer 13 and 14) and for 18S rRNA (GenBank accession no. AF207053, primer 15 and 16) (Supplementary Table S1) were designed using Primer Express 1.0 software (Life Technologies Inc.). Quantitative real-time RT-PCR was performed in technical triplicate and all samples were normalized to the data of 18S rRNA as an internal control.

### Extractions of α-guaiene and (−)-rotundone from grape tissues

α-Guaiene and (−)-rotundone were extracted as previously described (Takase *et al.*, 2015) with slight modification. Grape berry tissue was frozen and then pulverized in liquid nitrogen using a mixer mill (MM 400, Retsch, Haan, Germany). After adding 50 μl of ^2^H_5_-rotundone (50 μg l^-1^) as the internal standard, the powder (1g, fresh weight) was extracted with 5ml of n-pentane:ethyl acetate (9:1, v/v) by shaking for 1h in a shaker at room temperature. The extract was centrifuged at 3000rpm for 10min. The organic solvent was carefully removed by nitrogen gas purging. The residue was dissolved in 500 μl of ethanol, to which 4.5ml of aqueous tartrate buffer (pH 3.2) was added thereafter. The prepared grape samples were subjected to SBSE and GC-MS/MS analysis.

### Phylogenetic analysis

Phylogenetic analysis was performed using the entire predicted amino acid sequences of *V. vinifera* putative CYP71BE family proteins and related terpene-modifying P450 proteins from the GenBank database. Sequence alignments were generated on the basis of comparison of the amino acid sequences using the ClustalW program (Thompson *et al.*, 1994) with the following values: 10 for gap opening penalty and 0.1 for gap extension penalty in pairwise alignment; 10 for gap opening penalty and 0.2 for gap penalty in multiple alignment; Gonnet for protein weight matrix; available residue-specific penalties; available hydrophilic penalties; 4 for gap separation distance; and 30% delay divergent cutoff. These alignments were adopted to construct neighbor-joining phylogenetic trees using MEGA 6.06 with the scope of all selected taxa, amino acid substitution type, Poisson model, uniform rates, homogeneous pattern among lineages and complete deletion for gaps or missing data treatment (Tamura *et al.*, 2013). The scale bar of 0.1 indicates a 10% change and each number shown next to the branches is the number of replicate trees in which the related taxa clustered in the bootstrap test with 1000 replicates.

## Results

### Isolation of putative sesquiterpene oxidase genes from *V. vinifera* cv. Syrah

**Fig. 1.**
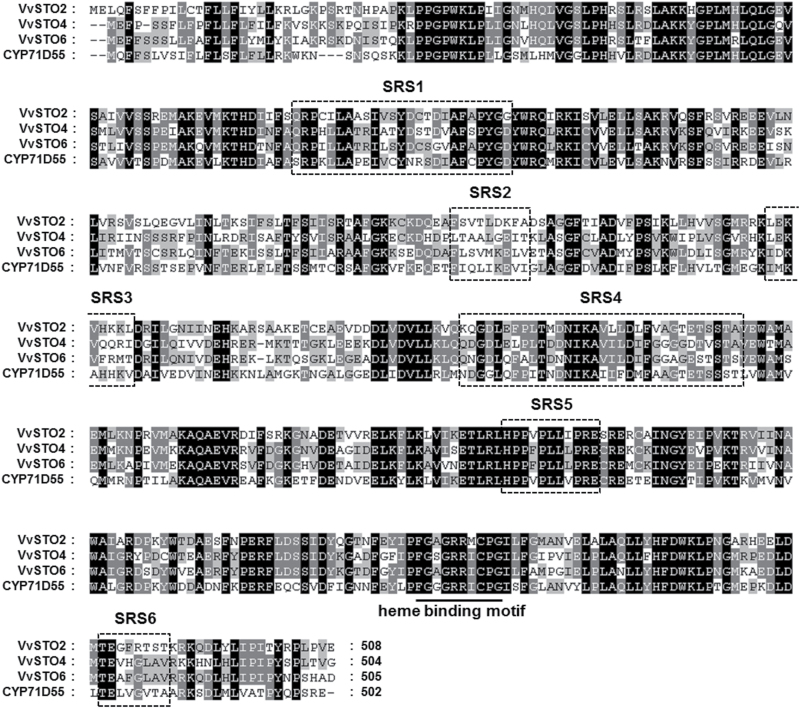
Amino acid sequence alignment of VvSTO2, VvSTO4, VvSTO6 and CYP71D55. Multiple sequence alignment was performed using ClustalW and visualized by the software Genedoc. Six of the putative SRS regions defined by Gotoh (Gotoh, 1992) are outlined and the heme-binding motifs are underlined and annotated. The black and gray shades indicate similar amino acids, respectively.

The premnaspirodiene oxygenase CYP71D55 can catalyze the successive reactions leading to the generation of solavetivone via the oxidation of premnaspirodiene at position C-2. It can also oxidize (+)-valancene at position C-2 to transform β-nootkatol (Takahashi *et al.*, 2007). Furthermore, it is a cytochrome P450 within the CYP71D subfamily. In particular, the successive oxidations of premnaspirodiene by CYP71D55 seem to be similar to our target reaction. Therefore, to identify putative sesquiterpene oxidases responsible for the oxidation of α-guaiene in grapevines, six CYP71D55-like genes (more than 50% identity to CYP71D55) were identified as candidates by homology sequencing with the premnaspirodiene oxygenase CYP71D55 from the 12-fold coverage genome sequence assembly of the grapevine cultivar Pinot Noir PN40024 (NCBI BioProject, Accession: PRJNA33471) (Jansen *et al.*, 2006; Jaillon *et al.*, 2007; Goremykin *et al.*, 2009). Comparison with the *V. vinifera* cytochrome P450s listed on the Cytochrome P450 Homepage (Nelson, 2009) suggests that these genes belong to the CYP71BE family. Its family overlaps with the very large CYP71D family, according to the information from the P450 nomenclature committee (care of Dr. Nelson). They were named *V. vinifera* sesquiterpene oxidases, *VvSTO1 to VvSTO6,* and corresponded to each reference sequence (RefSeq) from the 12-fold coverage grapevine genome sequence assembly listed in Supplementary Table S2. Among six uncharacterized candidate genes, three genes – *VvSTO2, VvSTO4* and *VvSTO6* – could be successfully isolated from the Syrah grape exocarp by PCR using specific primers. Three other genes could not be isolated in this study. The isolated cDNAs *VvSTO2, VvSTO4* and *VvSTO6* had the ORFs of 1527, 1515 and 1518 base pairs, and encoded the predicted proteins of 508, 504 and 505 amino acids, respectively. The alignment of their predicted amino acid sequences and CYP71D55 showed that they possessed the highly conserved motifs among the eukaryotic P450s, such as the heme-binding motif FxxGxRxCxG (Chapple, 1998; Ralston *et al.*, 2001) and the putative substrate recognition sites (SRSs) originally described by Gotoh (Gotoh, 1992) (Fig. 1). *VvSTO2, VvSTO4* and *VvSTO6* showed 53–58% sequence identity with CYP71D55 at the protein sequence level. These putative *V. vinifera* P450s, VvSTO2, VvSTO4 and VvSTO6, were subsequently assigned to CYP71BE5, CYP71BE1 and CYP71BE10 by the P450 nomenclature committee (Nelson, 2009), and their respective DDBJ accession numbers are LC055499, LC055500 and LC055501.

### Functional identification of *V. vinifera* cytochrome P450s by *in vitro* enzyme assay

To demonstrate the enzymatic function of the isolated genes, the yeast strain BJ2168 was transformed with the expression plasmid containing each ORF. *VvSTO2*, *VvSTO4* and *VvSTO6* expressions were confirmed by western blot analysis using an anti-V5-HRP antibody (Supplementary Fig. S1). The predicted molecular mass of VvSTO2, VvSTO4 and VvSTO6 fusion proteins with the V5 epitope were 61.4, 60.9 and 61.1kDa, respectively. For each fusion protein, the apparent band was detected in accord with the position of its predicted molecular mass.

**Fig. 2.**
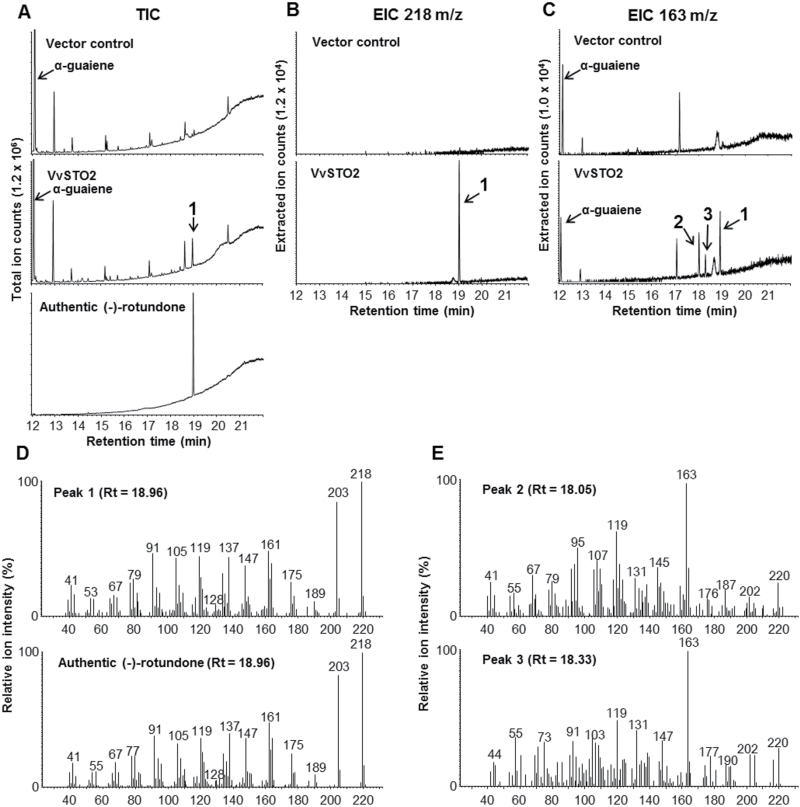
GC-MS analysis of enzymatic reaction products by *in vitro* assay of α-guaiene with VvSTO2. (A) Total ion chromatograms (TICs) of incubation mixtures resulting from *in vitro* assay of α-guaiene with vector control and recombinant VvSTO2, and of authentic (−)-rotundone. (B) Extracted ion chromatograms (EICs) at m/z 218 and (C) m/z 163 corresponding to each TIC of the same incubation mixtures resulting from *in vitro* assay of α-guaiene. (D) Mass spectra of peak 1 and authentic (−)-rotundone. (E) Mass spectra of peaks 2 and 3. The mass spectra of peak 2 and 3 were identical to those of (2R)-rotundol and (2S)-rotundol, respectively (Huang *et al.*, 2015a). Corresponding chromatograms were shown at the same scale as indicated in the vertical axis.

The microsomes from yeast cells expressing each of the candidate P450s were assayed *in vitro* with α-guaiene as the substrate and the reaction product was analyzed by GC-MS. No product was detected in assays using the microsomes from yeast cells containing an empty pYES vector (Fig. 2A). In contrast, the formation of a major reaction product by VvSTO2 catalysis (peak 1) was detected (Fig. 2A). The reaction product of peak 1 by VvSTO2 catalysis was apparently shown also in the extracted-ion chromatogram (EIC) of m/z 218 which was the molecular weight of (−)-rotundone (Fig. 2B). Furthermore, the EIC of m/z 163 showed two small peaks in addition to peak 1 (Fig. 2C). The retention time (Rt) and the mass spectrum of peak 1 matched those of authentic (−)-rotundone (Fig. 2A–D). Interestingly, the mass spectra of peaks 2 and 3 were identical to those of (2R)-rotundol and (2S)-rotundol (Fig. 2E), respectively (Huang *et al.*, 2015a). Additionally, VvSTO4 and VvSTO6 produced several reaction products with α-guaiene as a substrate (Supplementary Fig. S2). Their unknown reaction products were presumed to correspond to mono-hydroxylated sesquiterpenes on the basis of a parent ion of 220 or 222 m/z in GC-MS analysis and were not identified. However, the mass spectra of these unknown compounds were apparently different from those of (2R)-rotundol and (2S)-rotundol (Huang *et al.*, 2015a). The absence of spontaneous oxidative reaction of α-guaiene to (−)-rotundone was confirmed in our *in vitro* assay through performing numerous repetitions with the microsomes from yeast cells containing an empty pYES vector, VvSTO4 or VvSTO6 for 2 to 48h.

### Substrate specificity and kinetic properties of the VvSTO2 protein

**Fig. 3.**
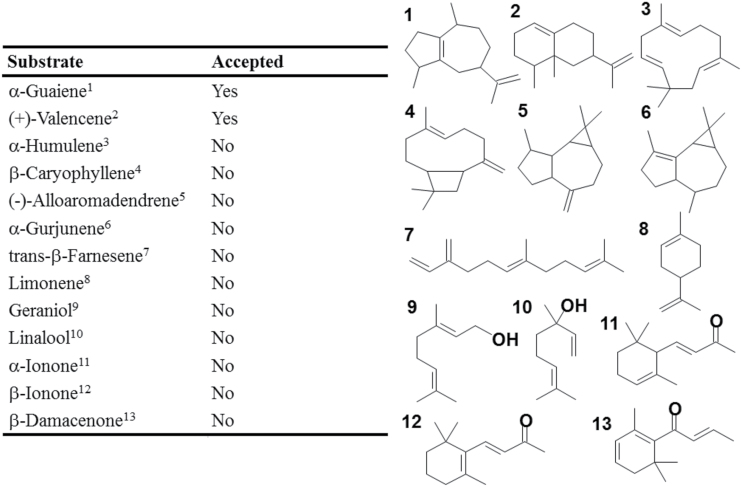
Substrate specificity of VvSTO2, with left-hand panel indicating whether VvSTO2 accepted each compound as a substrate. The right-hand side of the panel shows the structures of the corresponding numbered substrates.

The VvSTO2 protein was analyzed further for substrate specificity and kinetics, because it demonstrated the capability for rotundone synthesis from α-guaiene. The enzyme activity of the VvSTO2 protein was examined using other sesquiterpenes and monoterpenes as potential substrates, as listed in Fig. 3. The microsomes containing the VvSTO2 protein could use (+)-valencene as the substrate as well as α-guaiene. On the other hand, the VvSTO2 protein did not accept other sesquiterpenes, monoterpenes or C13 norisoprenoids as a substrate. As a result, β-nootkatol accumulated, as previously reported for the function of the CYP71D55 protein (Takahashi *et al.*, 2007) (Supplementary Fig. S3). However, other reaction products such as α-nootkatol and nootkatone were not detected.

**Fig. 4.**
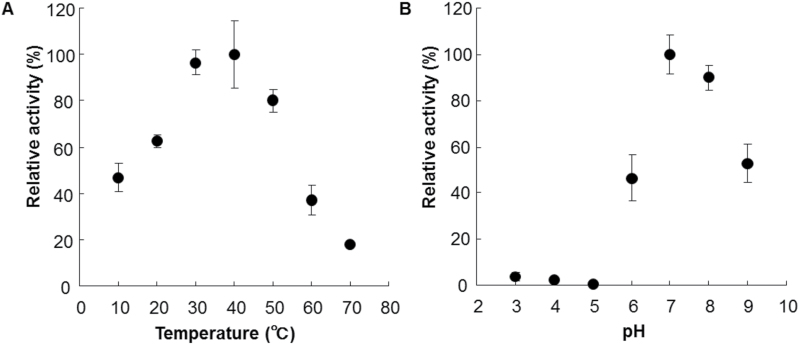
Enzymatic characterization of recombinant VvSTO2. (A) Temperature profile of VvSTO2 activity. (B) pH dependence of VvSTO2 activity. The buffers were citrate buffer (pH 3, 4, 5, 6), Tris-HCl buffer (pH 7, 8, 9). VvSTO2 activity was measured with α-guaiene as a substrate. Values are the mean ± standard deviation of three technical replicates.

The apparent *K*m values for the transformations of α-guaiene to (−)-rotundone and (+)-valencene to β-nootkatol were calculated from the Hanes-Woolf plot to be 30 μM and 35 μM, respectively (Supplementary Fig. S4). These low *K*m values are similar to those (7.4±1.2 μM to 52±2 μM) for other sesquiterpene oxidases that can transform (+)-valencene to β-nootkatol (Gavira *et al.*, 2013). As a result of the enzyme assays adding α-guaiene and (+)-valencene simultaneously, the production rates of (−)-rotundone and β-nootkatol were 82.7% and 73.3%, respectively, compared to the enzyme assays which added each substrate independently. These results suggest that α-guaiene and (+)-valencene can interfere with each enzyme reaction of VvSTO2, but the interference is relatively low. For transforming α-guaiene to (−)-rotundone, the optimum temperature was in the range from 30–40ºC and the optimum pH was in the range from 7.0–8.0 (Fig. 4).

### Patterns of the accumulation of α-guaiene, (−)-rotundone, and *VvSTO2* mRNA in grape berries

**Fig. 5.**
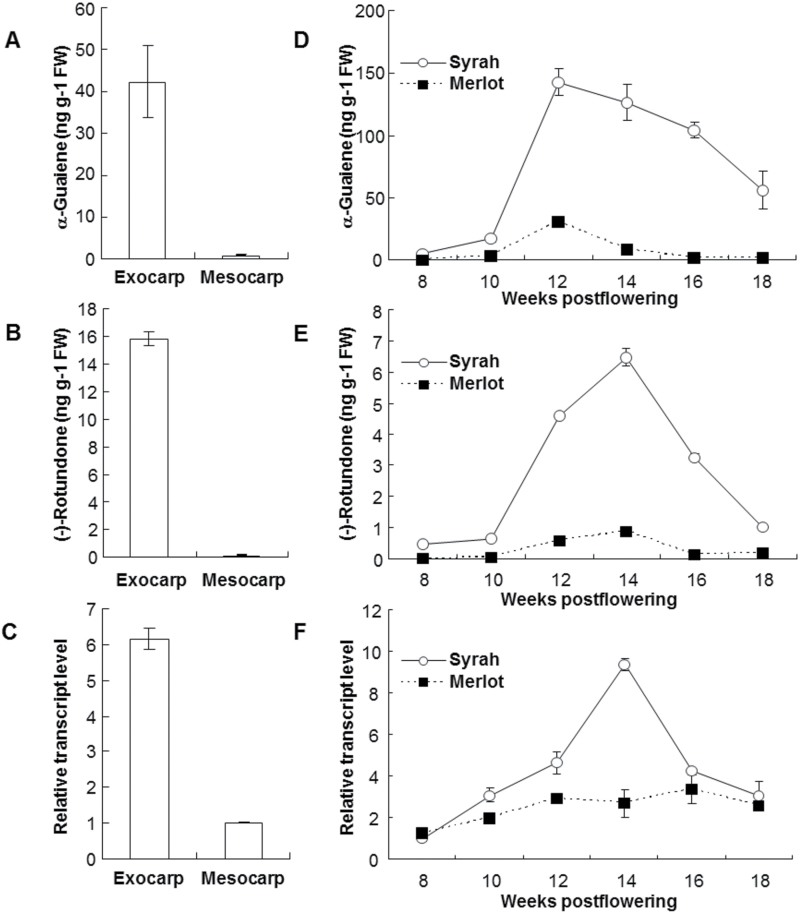
Patterns of α-guaiene and (−)-rotundone accumulation with relative transcript levels of *VvSTO2* mRNA in grape berries. (A) Concentration of α-guaiene, (B) concentration of (−)-rotundone and (C) relative transcript levels of *VvSTO2* mRNA in each tissue of Syrah grape berries. (D) Concentration of α-guaiene, (E) concentration of (−)-rotundone and (F) relative transcript levels of *VvSTO2* mRNA in Syrah and Merlot grape exocarp during grape maturation after flowering. The transcipt levels of *VvSTO2* mRNA were determined by the quantitative real-time RT-PCR. All samples were normalized using 18 rRNA as an internal control. The transcript levels in the mesocarp for the experiment in panel C and in the Syrah grape exocarp at 8 weeks postflowering for the experiment in panel F were set to 1 to calculate the relative transcript levels of *VvSTO2* mRNA, respectively. Values are the mean ± standard deviation of three technical replicates. FW, fresh weight.

The concentrations of α-guaiene and (−)-rotundone in grape berries were analyzed by GC-MS/MS using the SBSE method and *VvSTO2* transcript levels were quantified by real-time RT-PCR analysis. A previous report showed that (−)-rotundone is localized in the grape berry exocarp (Caputi *et al.*, 2011); however, the localization of α-guaiene was not clear. The tissue specificity of *VvSTO2* transcript levels and the concentration of α-guaiene and (−)-rotundone in various tissues of grape berries were investigated using mature Syrah grapes. The concentrations of α-guaiene and (−)-rotundone were higher in the exocarp than in mesocarp (flesh) (Fig. 5A, B). Consistent with this, the *VvSTO2* transcript levels in the exocarp were much higher than those in the mesocarp (Fig. 5C). These findings further support the observation that sesquiterpene biosynthesis and accumulation in grape berries is restricted to the exocarp (May *et al.*, 2013). Furthermore, the patterns of the accumulation of α-guaiene and (−)-rotundone and *VvSTO2* transcript levels in the exocarp during the grape maturation between 8 and 18 weeks postflowering were investigated, comparing the high-rotundone cultivar Syrah with the low-rotundone cultivar Merlot. At the same time, the general fruit components such as the total soluble solid and the titratable acidity during grape maturation were analyzed (Supplementary Fig. S5). The accumulation of α-guaiene in the Syrah grape exocarp reached a maximum at 12 weeks postflowering and then decreased (Fig. 5D). Interestingly, the accumulation of (−)-rotundone reached the maximum at 14 weeks postflowering, which is 2 weeks later than the accumulation of α-guaiene, and then decreased progressively (Fig. 5E). These results support the hypothesis that the biosynthesis of (−)-rotundone occurs following the accumulation of α-guaiene. In contrast, the concentrations of α-guaiene and (−)-rotundone in the Merlot grape exocarp was always much lower than those in the Syrah grape exocarp during grape maturation (Fig. 5D, E). Moreover, the patterns of *VvSTO2* transcript levels in the Syrah and Merlot grape exocarps were completely different and consistent with the patterns of (−)-rotundone accumulation during grape maturation (Fig. 5F). Taken together, these findings suggest that VvSTO2 is involved in the biosynthesis of (−)-rotundone in grape berries.

## Discussion

(−)-Rotundone is as one of the most important compounds significantly contributing to the spicy characteristic notably in wines and grapes, since its first identification in the red wine made from the grape cultivar Syrah in 2008 (Wood *et al.*, 2008). Although the factors affecting the accumulation of (−)-rotundone and its chemical formation via the oxidation of α-guaiene have been reported (Huang *et al.*, 2014), the mechanism of (−)-rotundone biosynthesis in plants has thus far remained unclear.

**Fig. 6.**
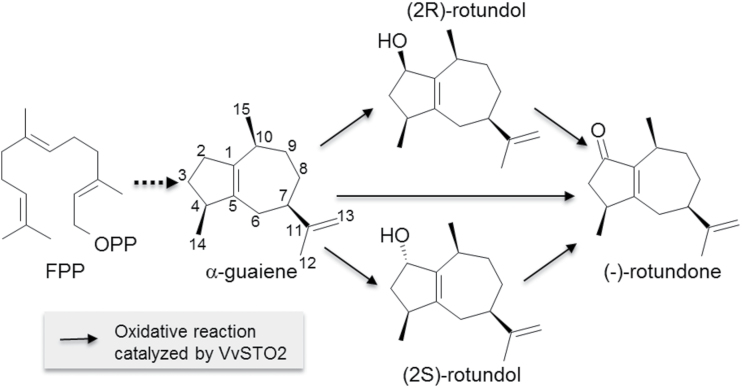
Proposed pathway for biosynthesis of (−)-rotundone. FPP, farnesyl diphosphate; OPP, diphosphate in the structure of FPP.

**Fig. 7.**
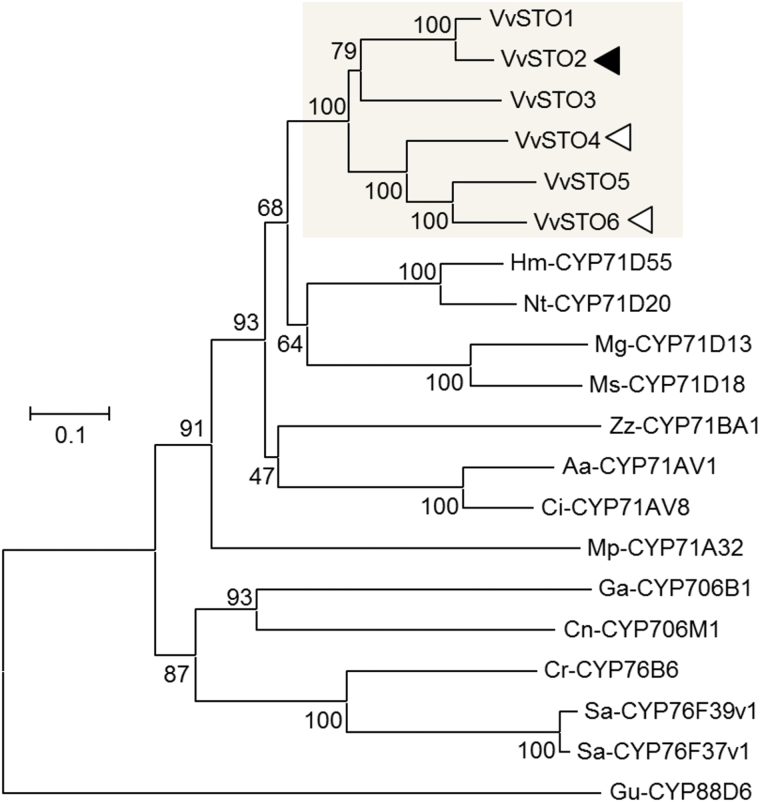
Phylogenetic tree of *V. vinifera* CYP71BE proteins and related terpene-modifying CYPs from various plants including *Artemisia annua* (Aa), *Catharanthus roseus* (Cr), *Cichorium intybus* (Ci), *C. nootkatensis* (Cn), *Gossypium arboreum* (Ga), *H. muticus* (Hm), *Mentha × gracilis* (Mg), *Mentha × piperita* (Mp), *Mentha spicata* (Ms), *N. tabacum* (Nt), *Santalum album* (Sa), *Zingiber zerumbet* (Zz). The neighbor-joining tree was generated using ClustalW and MEGA5. The numbers indicate the bootstrap value (%) from 1000 replications. The scale bar shows the amino acid substitution ratio. Gu-CYP88D6, *Glycyrrhiza uralensis* β-amyrin-11-oxidase (AB433179), was used as the outgroup. *V. vinifera* CYP71BE proteins identified in this study are shown in the gray box. The closed arrowhead indicates VvSTO2 having the activity of rotundone synthesis and the open arrowheads indicate the CYPs isolated, incapable of producing (−)-rotundone in this study.

To identify the key enzyme involved in the biosynthesis of (−)-rotundone, we performed screening with the homology sequence of characterized sesquiterpene oxidase from the 12-fold coverage genome sequence assembly of the grapevine cultivar Pinot Noir PN40024 (Jansen *et al.*, 2006; Jaillon *et al.*, 2007; Goremykin *et al.*, 2009). In this study, we identified a novel P450 enzyme, the α-guaiene 2-oxidase VvSTO2, from the grapevine cultivar Syrah, which can oxidize α-guaiene at position C-2, leading to the biosynthesis of (−)-rotundone (Fig. 6). VvSTO2, assigned as CYP71BE5, belongs to the CYP71BE subfamily of the CYP71 family within the CYP71 clan of enzymes. Moreover, the CYP71BE subfamily overlaps with the very large CYP71D subfamily. To the best of our knowledge, this study is the first to functionally characterize CYP71BE5 in this enzyme family. The CYP71 clan accounts for more than 50% of all plant CYPs. Although a huge diversity of their functions has been reported, including monoterpene and sesquiterpene oxidation in plant terpenoid metabolism (Nelson and Werck-Reichhart, 2011), plant CYPs capable of catalyzing α-guaiene to (−)-rotundone have never been functionally characterized. Phylogenetic analysis showed that VvSTO2 was closely related to other terpene-modifying CYPs, mostly enzymes belonging to the CYP71 family (Fig. 7).

Several sesquiterpene oxidases that can catalyze a multistep oxidation of sesquiterpene in plants have been reported in previous studies. For example, the amorphadiene oxidase CYP71AV1 from *Artemisia annua* catalyzes a three-step oxidation of amorpha-4,11-diene to artemisinic acid (Ro *et al.*, 2006), the 5-epi-aristolochene-1,3-dihydroxylase CYP71D20 from *Nicotiana tabacum* catalyzes a two-step oxidation of 5-epi-aristolochene to capsidiol (Takahashi *et al.*, 2005), the premnaspirodiene oxygenase CYP71D55 from *Hyoscyamus muticus* catalyzes a two-step oxidation of premnaspirodiene to solavetivone (Takahashi *et al.*, 2007), and the valencene oxidase CYP706M1 from *Cupressus nootkatensis* catalyzes a two-step oxidation of (+)-valencene to (+)-nootkatone (Cankar *et al.*, 2014). These reactions showed coincidentally the existence of some intermediate compounds such as mono-hydroxylated sesquiterpene. Similarly, VvSTO2 generated (−)-rotundone as the major reaction product with α-guaiene, and presumably (2R)-rotundol and (2S)-rotundol as the intermediate compounds to synthesize (−)-rotundone were found *in vitro* enzyme assays, although their peaks were at trace levels. These results suggest that this enzyme is involved in the enzymatic oxidation of α-guaiene to (−)-rotundone and can catalyze a one-step oxidation of α-guaiene to (−)-rotundone or a two-step oxidation via a rapid second oxidation from (2R)-rotundol and (2S)-rotundol to (−)-rotundone (Fig. 6). To verify our proposed biosynthesis pathway of (−)-rotundone, further investigation using the synthetic standards of (2R)-rotundol and (2S)-rotundol is required.

The α-guaiene 2-oxidase VvSTO2 could oxidize (+)-valencene at position C-2 to β-nootkatol as well as α-guaiene at position C-2 to (−)-rotundone (Supplementary Fig. S3). On the other hand, VvSTO2 did not accept other sesquiterpenes, monoterpenes or C13 norisoprenoids as substrates (Fig. 3). These findings suggest that α-guaiene 2-oxidase possesses a relatively narrow substrate specificity for α-guaiene and (+)-valencene. VvSTO2 exhibited high affinity for α-guaiene and (+)-valencene substrates with low *K*m values. Both *K*m values of VvSTO2 ranged at the same levels as those of other sesquiterpene oxidases capable of transforming (+)-valencene to β-nootkatol (Gavira *et al.*, 2013). Therefore, VvSTO2 might have the ability to act like α-guaiene 2-oxidase and/or (+)-valencene oxidase in grapevine. However, neither (+)-valencene nor β-nootkatol were identified in the Syrah and Merlot grape exocarps in this study. This might indicate that (+)-valencene and β-nootkatol are either contained at extremely low concentrations or are not contained in the grape exocarp. To the best of our knowledge, neither compound has been reported from the grape exocarp previously. These facts suggest that VvSTO2 plays a role as α-guaiene 2-oxidase rather than as (+)-valencene oxidase in the grape exocarp. In addition, regarding the regio-position of sesquiterpene that undergoes the oxidation by a CYP, the position C-2 of premnaspirodiene, the positions C-1, 2 and 3 of 5-epi-aristolochene, the positions C-2 and 12 of (+)-valencene, the position C-12 of germacrene A, the position C-12 of amorpha-4,11-diene and the position C-8 of α-humulene were reported (Takahashi *et al.*, 2005, 2007; Cankar *et al.*, 2011; Yu *et al.*, 2011). The oxidation by VvSTO2 at the regio-position C-2 of α-guaiene was firstly observed. Furthermore, VvSTO4 and VvSTO6 also showed the capacity to oxidize α-guaiene to as yet unknown compounds including two compounds produced in common by both enzymes such as the peak 4 and 5 (Supplementary Fig. S3), but (−)-rotundone, (2R)-rotundol and (2S)-rotundol were not produced by them. These particular similarities of enzyme properties of VvSTO4 and VvSTO6 may be due to their amino acid sequence identity (71%). These observations suggest that VvSTO2 has unique regio-specificity compared with other *V. vinifera* CYP71BE proteins. VvSTO4 and VvSTO6 showed 61–62% amino acid sequence identity with VvSTO2, therefore the unique modification of α-guaiene to produce (−)-rotundone by VvSTO2 may be characterized by the difference in amino acid residues. In previous studies, the site-specific mutation of plant CYPs showed that residues such as those within SRS regions may affect the substrate selectivity, reaction product specificity, kinetic properties and regio-specificity (Kahn *et al.*, 2001; Komori *et al.*, 2013; Takahashi *et al.*, 2005, 2007). To fully understand the substrate specificity and regio-specificity of VvSTO2, further functional and structural characterization by a site-specific mutation study is required.

Quantitative real-time RT-PCR analyses showed that *VvSTO2* transcript levels in the exocarp were higher than those in the mesocarp in accordance with the localization of (−)-rotundone in grape berries (Fig. 5A–C). Additionally, α-guaiene was also detected in the exocarp at an extremely high concentration. These findings suggest that the accumulation of (−)-rotundone is regulated by the expression of *VvSTO2* as well as the biosynthesis of α-guaiene.

*VvSTO2* expression in the Syrah grape exocarp during grape maturation showed a similar pattern to the accumulation of (−)-rotundone and α-guaiene (Fig. 5D–F). Additionally, *VvSTO2* expression during grape maturation was considerably higher in Syrah grape exocarp compared to Merlot grape exocarp, consistent with the patterns of α-guaiene and (−)-rotundone accumulation. Thereby, it may explain why (−)-rotundone concentration of Syrah grape was higher than that of Merlot grape. These functional analyses findings of VvSTO2 suggest that it plays a critical role as a α-guaiene 2-oxidase in the biosynthesis of (−)-rotundone in grapevines and that the accumulation of (−)-rotundone is also controlled by the biosynthesis of α-guaiene. Chemical oxidation of α-guaiene to (−)-rotundone may occur non-enzymatically according to previous reports (Huang *et al.*, 2014), although it was not observed in our *in vitro* assay. These facts suggest that the enzymatic conversion of α-guaiene is more rapid than the non-enzymatic conversion in solution. Therefore it is more likely that the biosynthesis of (−)-rotundone from α-guaiene occurs enzymatically in grapevines, although some chemical oxidation cannot be ruled out. To fully determine the function of VvSTO2 *in vivo* in grapevine, transient expression assays such as over-expression studies of *VvSTO2* in grapevine tissues not containing (−)-rotundone or containing it at low concentration, if necessary by feeding α-guaiene substrate, and knock-down studies of *VvSTO2* in the high-rotundone cultivar Syrah, are required.

Sesquiterpenes are biosynthesized primarily via the mevalonate pathway operating in the cytosol and 2-C-methyl-D-erythriol-4-phosphate operating in plastids, and various sesquiterpene synthases contribute to the diversification of sesquiterpenes in plants (Chen *et al.*, 2011). Several sesquiterpene synthases such as (−)-valencene synthase and (−)-germacrene D synthase have been isolated from grapevines (Lucker *et al.*, 2004; Martin *et al.*, 2009). Moreover, 69 putatively functional terpene synthase (TPS) genes, including 13 characterized sesquiterpene synthase genes belonging to the TPS-a family, were identified from the 12-fold coverage genome sequence assembly of the grapevine cultivar Pinot Noir (Martin *et al.*, 2010). However, sesquiterpene synthase responsible for α-guaiene synthesis was not identified in grapevines, although a few enzymes producing α-guaiene as one of the major products were found in agarwood and patchouli (Faraldos *et al.*, 2010; Kumeta and Ito, 2010). Recent studies have proposed that genes such as TPS and CYP involved in terpene biosynthetic pathways are organized as metabolic gene clusters and are located adjacent to each other in *Arabidopsis* (Field and Osbourn, 2008; Field *et al.*, 2011), oat (Mugford *et al.*, 2013) and rice (Shimura *et al.*, 2007; Wilderman *et al.*, 2004). Similarly, in eudicots and monocots, the unique patterns of TPS and CYP gene assembly were observed, indicating that TPS genes primarily pair with CYP71 clan genes, notably with the members of the CYP71 family (Boutanaev *et al.*, 2015). Interestingly, *VvSTO2* is clustered with two other putative *CYP71BE*s (*VvSTO1* and *VvSTO3*; 90% and 66% identity with *VvSTO2* at the amino acid sequence level, respectively) in a length of 36kb on chromosome 19. In addition, ten putative sesquiterpene synthases were also located on chromosome 19 (Martin *et al.*, 2010), although their locations were relatively far from *VvSTO2*, in lengths ranging from 2.8 to 6.9Mb. The genetic organization may give an important clue to the identity of novel enzymes such as α-guaiene synthase and homologs of VvSTO2, relative to the biosynthesis of (−)-rotundone in grapevines.

Here, we successfully identified the α-guaiene 2-oxidase VvSTO2, which produces (−)-rotundone from α-guaiene via the (−)-rotundone biosynthetic pathway. *VvSTO2* expression analysis gave strong evidence for VvSTO2 as a key enzyme in the biosynthesis of (−)-rotundone of grape berries. Moreover, we demonstrated that the accumulation of (−)-rotundone is regulated by the biosynthesis of α-guaiene as its precursor in grapevines. Currently, the elucidation of environmental factors and regional characteristics that affect the accumulation of (−)-rotundone is in progress. However, it is extremely difficult to evaluate and to obtain evidence for the effect of each factor due to the lack of information on the target gene in the biosynthesis of (−)-rotundone. The present study’s discoveries might contribute to a more profound understanding of the mechanism of (−)-rotundone biosynthesis in grapevines and, by providing target markers as a useful tool, could promote investigations that elucidate the mechanism by which the regional character, that is *terroir*, affects the accumulation of (−)-rotundone. Additionally, while (−)-rotundone is found in various important herbs and spices such as black and white pepper, oregano, basil, thyme, marjoram and rosemary (Wood *et al.*, 2008), the α-guaiene 2-oxidase CYP71BE5 capable of synthesizing (−)-rotundone is isolated from only grapevines in this study. The genetic information of CYP71BE5 could serve as a powerful tool to isolate the common functional CYPs from other plants.

## Supplementary data

Supplementary data are available at *JXB* online.

Table S1. Specific primers used in this work.

Table S2. Putative CYP71BE family genes from the 12-fold coverage genome sequence assembly of the grapevine cultivar Pinot Noir PN40024.

Figure S1. Western blot analyses of recombinant *V. vinifera* P450s.

Figure S2. GC-MS analysis (total ion chromatogram) of enzymatic reaction products using α-guaiene with recombinant VvSTO4 and VvSTO6.

Figure S3. GC-MS analysis (total ion chromatogram) of enzymatic reaction products using (+)-valencene with VvSTO2.

Figure S4. Hanes-Woolf plots of recombinant VvSTO2 for α-guaiene and (+)-valencene.

Figure S5. Analysis of general fruit components during grape maturation.

Supplementary Data
