# Supplementary material for: Cytochrome P450 CYP71BE5 in grapevine (Vitis vinifera) catalyzes the formation of the spicy aroma compound (−)-rotundone
Source: J Exp Bot. 2015 Nov 20;67(3):787–98. doi: 10.1093/jxb/erv496 (PMC4737078; doi:10.1093/jxb/erv496)
Supplement: Supplementary Data [file supp_67_3_787__index.html]

Cytochrome P450 CYP71BE5 in grapevine (Vitis vinifera) catalyzes the formation of the spicy aroma compound (−)-rotundone — Cytochrome P450 CYP71BE5 in grapevine (Vitis vinifera) catalyzes the formation of the spicy aroma compound (−)-rotundone — Supplementary Data 

# Cytochrome P450 CYP71BE5 in grapevine (*Vitis vinifera*) catalyzes the formation of the spicy aroma compound (−)-rotundone

## Supplementary Data

Data files

- Supplementary\_Tables\_S1\_S2\_Figures\_S1\_S5.pdf - Supplementary Data
