# Supplementary material for: Cytochrome P450 CYP71BE5 in grapevine (Vitis vinifera) catalyzes the formation of the spicy aroma compound (−)-rotundone
Source: J Exp Bot. 2015 Nov 20;67(3):787–98. doi: 10.1093/jxb/erv496 (PMC4737078; doi:10.1093/jxb/erv496)
Supplement: Supplementary Data [file supp_erv496_Supplementary_Tables_S1_S2_Figures_S1_S5.pdf]

Title:
Cytochrome P450 CYP71BE5 from grapevine catalyzes the formation of the spicy aroma compound, (-)-rotundone

Authors:
Hideki Takase, Kanako Sasaki, Hideyuki Shinmori, Akira Shinohara, Chihiro Mochizuki, Hironori Kobayashi, Gen Ikoma, Hiroshi Saito, Hironori Matsuo, Shunji Suzuki, and Ryoji Takata

Table S1. Specific primers used in this work.

Primer No.	Orientation	Sequence (5' to 3')
1	F	CCTCTCTACTGGTCATCTCTTTTCTCC
2	R	GCATTGCATTTCAGATAAATAGCATCG
3	F	CAAAGATGGCGCTTGTGCTGAG
4	R	CATTACATCACAGCGGCTTCTG
5	F	GGTATCTCTTCTAAGAAACCTCTAACA
6	R	GCATTGATCCAAATACAAAACACCCA
7	F	ATGGAGCTCCAATTCTCCTTCTTCC
8	R	TTCAACAGGCAGAGGACGATAAGTAATG
9	F	ATGGAGTTTCCCTCTTCTTTCCTC
10	R	CCCAACAGTCAATGGACTATAAGG
11	F	ATGGAGTTCTTCTCCTCTTCTC
12	R	ATCAGCATGCGATGGATTATAAG
13	F	CTACTGGTCATCTCTTTTCTCCTC
14	R	ACTTCGGAGCAGGATGGTTG
15	F	CGAAAGCATTTGCCAAGGAT
16	R	CCTGGTCGGCATCGTTTATG

Table S2. Putative CYP71BE family genes from the 12 fold coverage genome sequence assembly of the grapevine cultivar Pinot Noir PN40024.

Clone Name	Accession No. of NCBI RefSeq		Homology with CYP71D55
	mRNA	Protein	
VvSTO1	XM_010646245	XP_010644547	56%
VvSTO2	XM_010646246	XP_010644548	58%
VvSTO3	XM_010646430	XP_010644732	50%
VvSTO4	XM_010654905	XP_010653207	55%
VvSTO5	XM_010657568	XP_010655870	52%
VvSTO6	XM_010657579	XP_010655881	53%

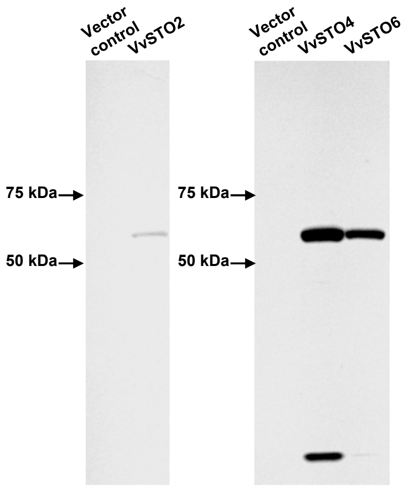

Figure S1. Western blot analyses of recombinant *V. vinifera* P450s. The recombinant proteins (10 μg) were loaded for each lane, and detected by the western blotting with a mouse monoclonal anti-V5-HRP antibody (Invitrogen) and an ECL Prime Western Blotting Detection Reagent (Amersham). The predicted molecular mass of VvSTO2, VvSTO4 and VvSTO6 fusion protein with the V5 epitope were 61.4, 60.9 and 61.1, respectively.

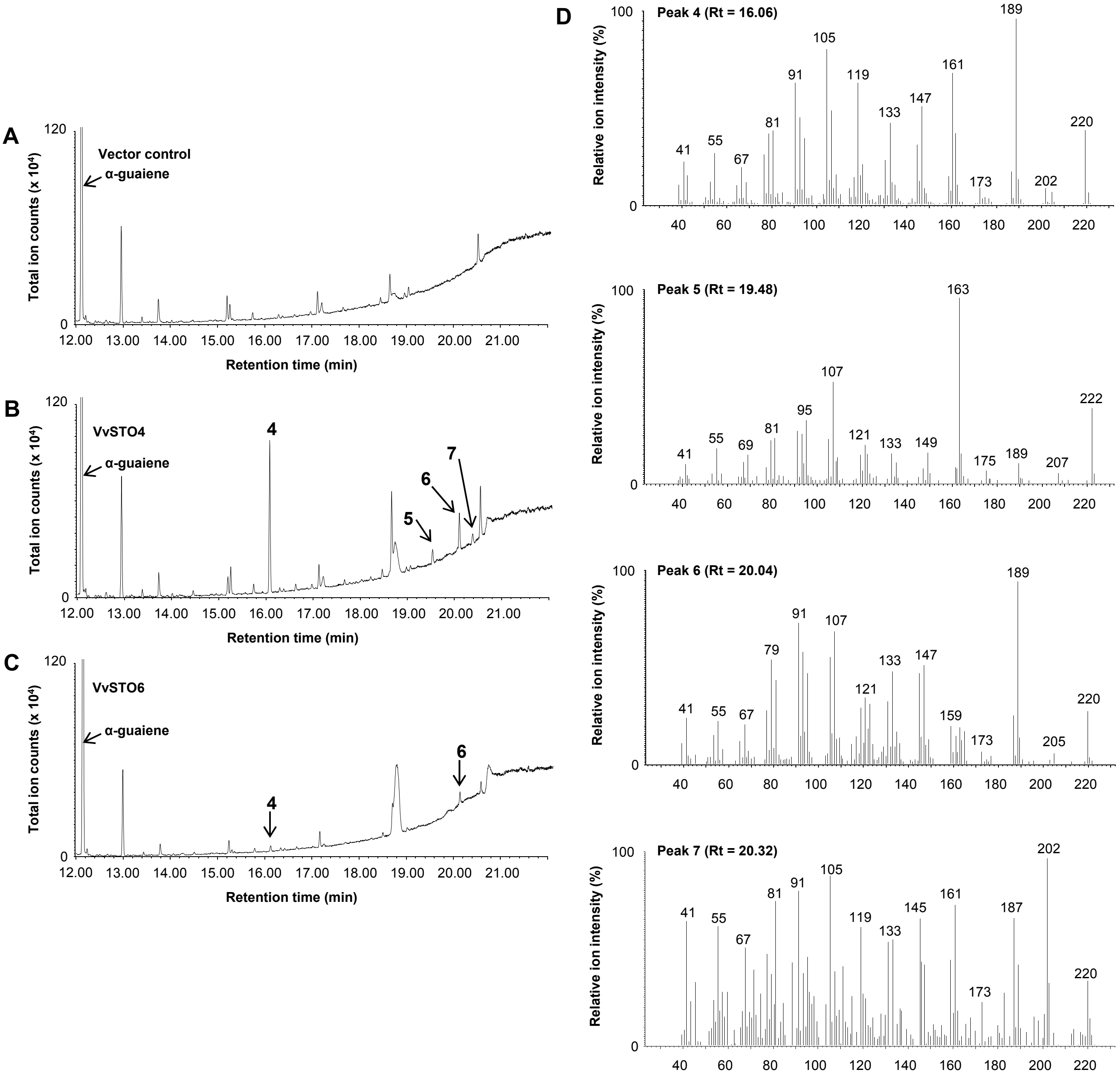

Figure S2. GC-MS analysis (total ion chromatogram) of the enzymatic reaction products using α-guaiene with recombinant VvSTO4 and VvSTO6. (A) Incubation mixture resulting from *in vitro* assay of α-guaiene with vector control, (B) recombinant VvSTO4 and (C) VvSTO6. (D) The mass spectra of unknown peaks 4 to 7.

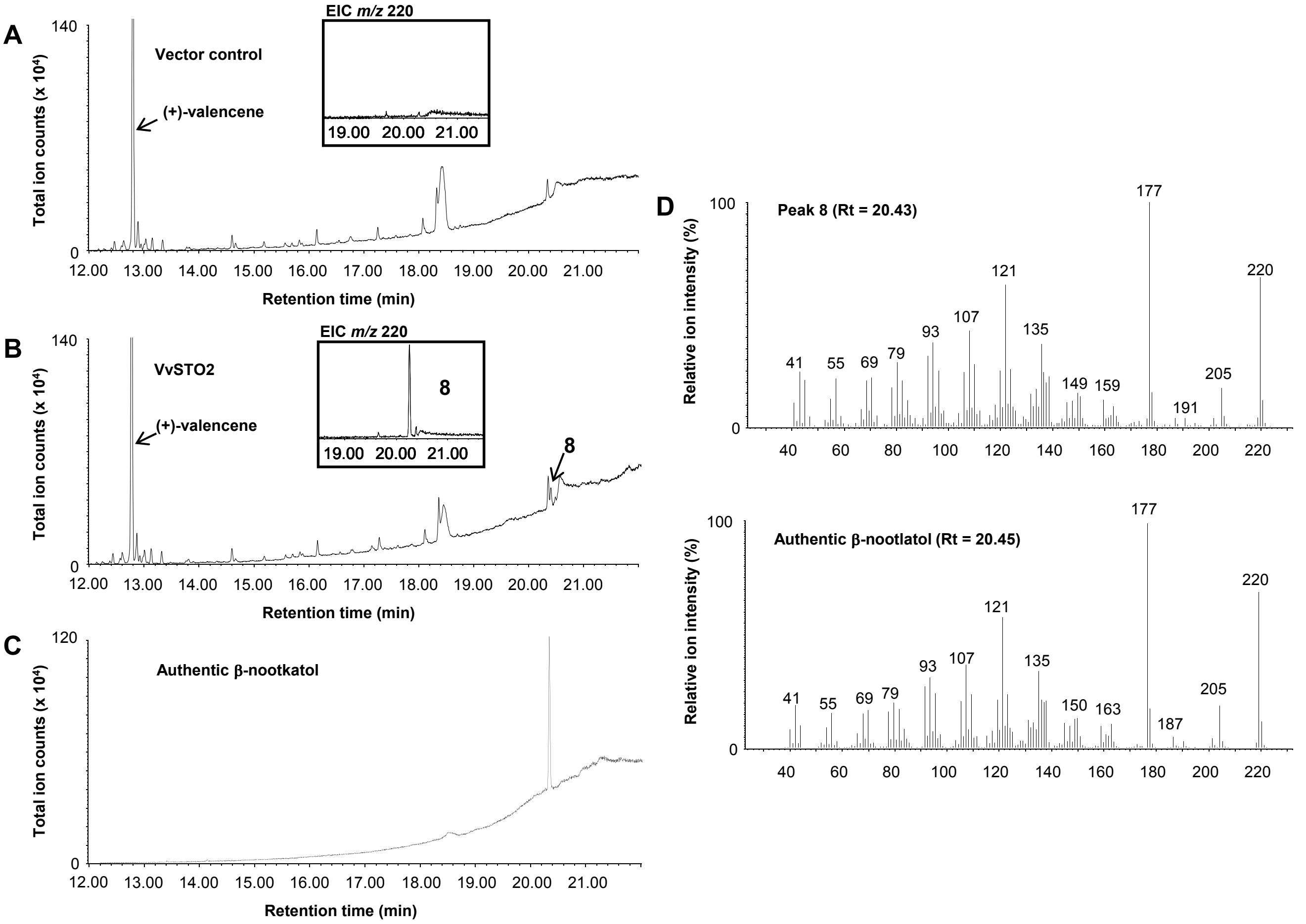

Figure S3. GC-MS analysis (total ion chromatogram) of the enzymatic reaction products using (+)-valencene with VvSTO2. (A) Incubation mixture resulting from *in vitro* assay of (+)-valencene with vector control, and (B) recombinant VvSTO2. Inserts show enlargements of the extracted ion chromatograms at m/z 220 corresponding to retention times of 19.0 to 21.0 min. (C) The chromatogram of authentic β-nootkatol. (D) The mass spectra of peak 8 and that of authentic β-nootkatol.

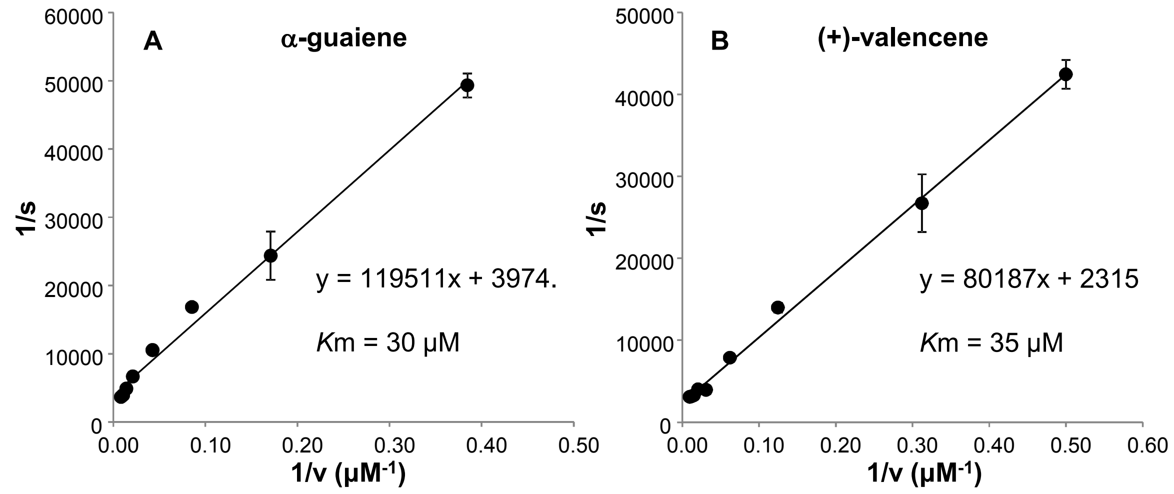

Figure S4. Hanes-Woolf plots of recombinant VvSTO2 for (A) α-guaiene and (B) (+)-valencene. Reaction products were analyzed by GC-MS using SBSE method. 1/s is expressed as [(area counts of reaction product) (area counts of internal standard)$^{-1}$ min^{-1}]$^{-1}$. Values are the mean ± standard deviation of three technical replicates.

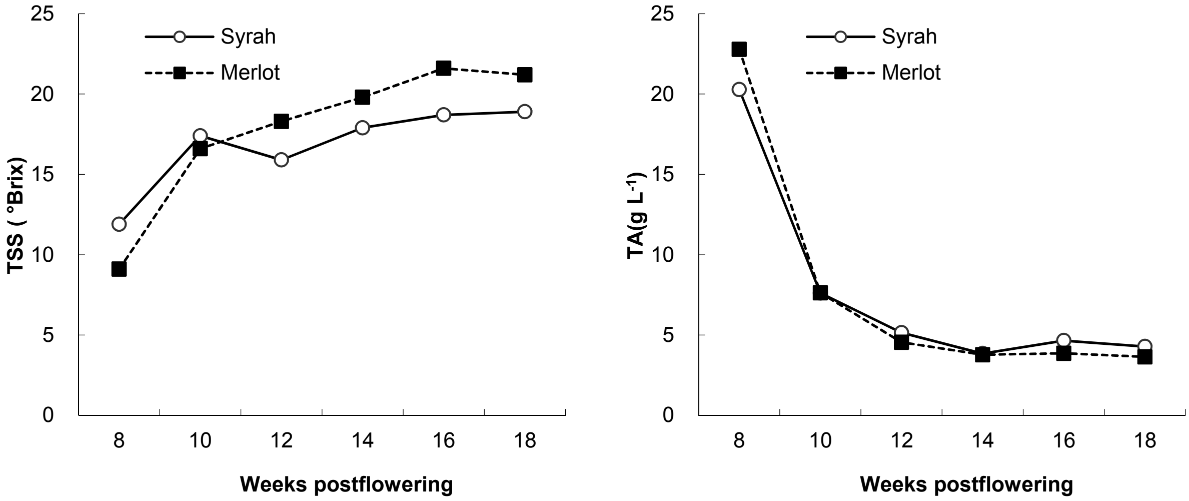

Figure S5. Analysis of general fruit components during grape maturation. (A) Total soluble solid (TSS). (B) Titratable acidity (TA). For these analyses, grape juice was prepared by homogenizing grape berries in the mill followed by pressing to 60% of the total berry weight. TSS in juice was measured with a refractometer (Pocket PAL-1, ATAGO Co., Tokyo, Japan) and expressed as ºBrix. TA was determined by adding 10 mL of distilled water to 10 mL of juice and subjecting the solution to neutralization titration using 0.1 N NaOH. TA was expressed as g tartaric acid/L. Véraison occurred between 8 and 9 postflowering.
